# Bitter melon extract mitigates heterocyclic aromatic amine formation in chicken thigh meat

**DOI:** 10.1002/fsn3.4085

**Published:** 2024-03-07

**Authors:** Damla Gumus, Arife Macit, Bengu Guzel, M. Merve Tengilimoglu‐Metin, Mevlude Kizil

**Affiliations:** ^1^ Department of Nutrition and Dietetics, Faculty of Health Sciences Hacettepe University Ankara Turkey

**Keywords:** antioxidant, bitter melon, chicken, heterocyclic aromatic amines, marination, *Momordica charantia* L.

## Abstract

The purpose of the present research was to study the impact of bitter melon extract (BME) on the generation of heterocyclic aromatic amines (HAAs) in chicken thigh meat. Raw chicken samples were marinated overnight with various levels (0%, 0.5%, and 1%) of BME, and pan‐fried at 150, 200, and 250°C for a total of 10 min. IQx, IQ, MeIQx, MeIQ, 7,8‐DiMeIQx, 4,8‐DiMeIQx, PhIP, AαC, and MeAαC were detected in quantities that varied according to the cooking temperature and the concentration of BME. Notably, IQx, MeIQx, MeIQ, 7,8‐DiMeIQx, 4,8‐DiMeIQx, and AαC levels were reduced through the application of the marinade. Cooking at higher temperatures led to elevated levels of total HAAs. Total HAA levels were 0.98 ± 1.12 ng/g, 3.82 ± 2.12 ng/g, and 6.25 ± 3.35 ng/g in samples cooked at 150, 200, and 250°C, respectively (*p* < .01). BME demonstrated its effectiveness in mitigating total HAA levels, showing reductions ranging from 25.9% to 69.9%. The most effective concentration of BME in reducing total HAAs was 1% for all cooking temperatures, which might be attributed to its antioxidant activity. These results carry substantial implications for potentially incorporating natural extracts such as BME into chicken products as a viable strategy to reduce HAAs, thus enhancing the safety and quality of meat products.

## Graphical abstract

The impact of bitter melon extract (BME) on heterocyclic aromatic amine (HAA) formation in chicken thigh meat during pan‐frying at various temperatures has been investigated. Higher cooking temperatures led to increased total HAA levels, while marination with BME significantly mitigated HAA formation, demonstrating reductions ranging from 25.9% to 69.9%.

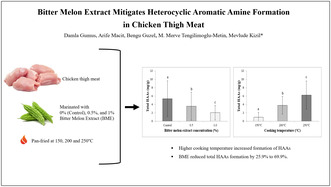

## 1 INTRODUCTION

The application of heat during meat preparation offers several advantageous outcomes, encompassing the mitigation of microbial hazards, improving nutrient digestibility, reduction of antinutritional components, augmentation of flavor and palatability, and extension of product shelf life (Geng et al., 2023; Suleman et al., 2020). Nevertheless, concurrent with these sought‐after effects, thermal treatment may also yield undesirable consequences, notably the depletion of certain nutrients, the occurrence of compounds adverse to sensory attributes, and the generation of carcinogenic and mutagenic compounds (Suleman et al., 2020). Among the carcinogenic and mutagenic compounds generated in cooked meats, a prominent class includes heterocyclic aromatic amines (HAAs). HAAs originate from chemical reactions involving amino acids, sugars, creatine, or creatinine within muscle‐based food products, including beef, chicken, pork, and fish, when subjected to cooking temperatures exceeding 100°C (Kang et al., 2022). As of the present, a total of 30 distinct HAAs have been identified, and these HAAs can be classified into two primary categories: polar (thermic) HAAs and nonpolar (pyrolytic) HAAs. Polar HAAs are generated as a result of the Maillard reaction, while nonpolar HAAs primarily originate from the thermal degradation of tryptophan and glutamic acid (Bulanda & Janoszka, 2022). The Maillard reaction incorporates free radicals such as pyridine and pyrazine radicals, along with reactive carbonyl structures, thereby leading to the proposition of both the free radical pathway and the carbonyl pathway as potential mechanisms for HAA synthesis (Dong et al., 2020; Zamora & Hidalgo, 2020). Chicken meat serves as a source of dietary HAAs. Despite typically showing lower HAA levels than certain other meat varieties, its widespread consumption significantly contributes to HAA intake within the diet (Geng et al., 2023). Widely consumed across diverse geographical regions globally (Lytou et al., 2017), chicken meat is renowned for its protein content and essential nutrients, including amino acids, vitamins, and minerals. Additionally, it contains reduced levels of total and saturated fat, as well as cholesterol, compared to red meat (Çapan & Bağdatli, 2021).

A multitude of experimental investigations have unveiled the pronounced mutagenic and carcinogenic proclivity of HAAs. These compounds possess the potential to induce mutations at specific genomic sites, effect deletions and insertions, and disrupt the hydrogen bonding patterns within the DNA molecule (Di et al., 2023; Geng et al., 2023; Yu et al., 2023). Considering the adverse health consequences attributed to HAAs, it becomes crucial to reduce their generation in cooked meat products, thereby mitigating potential health risks arising from dietary exposure. Consequently, there has been an increasing interest in formulating approaches to inhibit HAA formation, resulting in the development of various strategies in this regard (Geng et al., 2023). Prominent among these strategies are the alteration of cooking parameters and the incorporation of ingredients such as herbs, spices, and extracts derived from plants and fruits, which pose inhibitory effects during the HAA formation (Oz et al., 2023; Teng et al., 2023).

In recent years, the exploration of natural bioactive compounds as potential enhancers of food safety and nutritional quality has garnered significant attention (Geng et al., 2023). Bitter melon (BM) or bitter gourd (*Momordica charantia* L.), a plant of tropical origin, has emerged as a subject of particular interest due to its bioactive profile, which includes phytochemicals such as flavonoids, polyphenols, and alkaloids including momordicin, charantin, vicine, gentisic acid, catechin, chlorogenic acid etc (Ahmed et al., 2017; Psilopatis et al., 2023). Extensive research has underscored the various health‐promoting attributes of BM, ranging from its antidiabetic, antiviral, and anti‐inflammatory properties to its role as an antioxidant and antimicrobial agent (Gao et al., 2023; Jat et al., 2023). Considering the presence of bioactive compounds in BM and their well‐documented positive effects on health, previous studies have proposed the potential utilization of BM as an antioxidant and antibacterial agent in a diverse range of meat products (Jabeen & Khanum, 2017; Kuley et al., 2019; Siddique et al., 2023; Singh, 2013; Yavuzer et al., 2021). Despite the documented protective attributes of BME in various food applications (Kuley et al., 2019; Yavuzer et al., 2021), its specific role in mitigating HAA formation during cooking processes has not been thoroughly examined. Therefore, our study aims to address this research gap by investigating the potential influence of BME on HAA formation, providing valuable insights into its broader implications for food safety and quality.

In response to the recognized health risks associated with HAAs in cooked meats and the growing interest in natural antioxidants as potential mitigating agents, this study endeavors to elucidate the efficacy of BME as a natural agent in reducing the formation of HAAs in chicken meat. The focus on BME was driven by its rich array of bioactive compounds, and BM's historical significance in traditional medicine, coupled with its versatile culinary applications. Accordingly, our research is focused on investigating the mitigating impact of marinades containing varying concentrations of BME on the formation of HAAs in chicken thigh meat subjected to pan‐frying at different temperature conditions. By examining the effects of BME on HAA formation under controlled experimental conditions, this study aims to contribute to the scientific understanding of the potential role of natural antioxidants, such as BME, in promoting food safety and quality in cooked meat products.

## 2 MATERIALS AND METHODS

### 2.1 Raw materials

Fresh raw boneless skinless chicken thigh meats were bought from a local store (Ankara, Turkey), and brought to the laboratory on ice. BME was supplied by a commercial firm specializing in food supplements (Balen) located in Ankara, Turkey.

### 2.2 Chemicals and standards

The HAA standards of IQ (2‐amino‐3‐methylimidazo [4,5‐f] quinoline), IQx (2‐amino‐3‐methylimidazo [4,5‐f] quinoxaline), MeIQ (2‐amino3,4‐dimethylimidazo [4,5‐f] quinoline), MeIQx (2‐amino‐3,8‐dimethylimidazo [4,5‐f] quinoxaline), 7,8‐DiMeIQx (2‐amino‐3,7,8‐trimethylimidazo [4,5‐f] quinoxaline), 4,8‐DiMeIQx (2‐amino‐3,4, 8‐trimethylimidazo [4,5‐f] quinoxaline), 4,7,8‐TriMeIQx (2‐amino3,4,7,8‐tetramethylimidazo [4,5‐f] quinoxaline), Trp‐P‐2 (3‐amino‐1‐methyl‐5H‐pyrido [4,3‐b] indole), PhIP (2‐amino‐1‐methyl‐6‐phenylimidazo [4,5‐b] pyridine), AαC (2‐amino‐9H‐pyrido [2,3‐b] indole), and MeAαC (2‐amino‐3‐methyl‐9H‐pyrido [2,3‐b] indole) were purchased from Toronto Research Chemicals (Toronto, Canada). Reagents utilized for the extraction and analysis of HAAs were obtained from Merck (Germany). 4,7,8‐TriMeIQx was employed as the internal standard in the analysis of HAAs. Standard solutions of HAAs were prepared using methanol. HPLC analysis employed chemicals and solutions of HPLC grade.

### 2.3 Sample preparation and cooking procedures

An initial sensory evaluation was conducted by researchers to identify suitable levels of BME that would not adversely impact the specific sensory attributes of cooked chicken thigh. After assessing general acceptability based on visual and flavor properties, concentrations of the extract at 0.5% and 1% were determined to be appropriate. In the preparation of the marinades, 0.5 g and 1 g of BME were added per 100 g of marinade. The BME was dissolved in distilled water at a ratio of 1/10 (w/v) using a vortex. A control group with 0% BME concentration was included for comparison.

The chicken samples were subjected to random allocation into nine groups, considering variations in cooking temperature (150, 200, and 250°C) and BME concentration (0%, 0.5%, and 1%). Prior to preparation, visible fat was removed from all samples, and subsequently, the samples were uniformly sliced into fillets, each measuring 1 cm in thickness and weighing 100 g. This standardization ensured consistent size and shape across all samples. Each treatment group involved the marination and cooking of two separate chicken thighs. The marinade blend was uniformly spread over both sides of the chicken samples. The samples were placed in marinade solutions and kept submerged at 4°C overnight, aiming to maximize the penetration and overall efficacy of BME.

The cooking duration, established based on preliminary experiments, was set at 5 min per side. The cooking process was conducted using a new nonstick pan coated with Teflon. The pan had no scratches, dents, or other damage to the coating, ensuring the maintenance of its nonstick properties. After cooking each sample, the residues in the pan were removed, the pan was washed and dried, and the subsequent sample was cooked after this cleaning process. Samples were cooked without the addition of oil at three different temperatures resulting in a total cooking time of 10 min. Surface temperature was continuously monitored throughout the entire cooking process and adjusted by utilizing a surface thermometer (Testo 905‐T2). The internal temperature measurement was carried out using a probe thermometer (Testo 905‐T1). Following the cooking, the samples were subjected to ambient temperature for cooling and homogenized using a blender and were subsequently stored at a temperature of −20°C until laboratory analyses.

### 2.4 Proximate composition, pH analysis, and cooking loss

The proximate analysis of raw and cooked samples, encompassing evaluations of protein, lipid, ash, and moisture content, was conducted in accordance with the established protocols of AOAC (Horwitz, 2010). The pH values of the samples were analyzed employing a calibrated digital pH meter (Isolab GmbH, Germany). To ascertain the cooking loss, the samples were weighed both prior to and postcooking, and the findings were presented as a percentage.

### 2.5 HAA extraction and HAA analysis

The analysis of HAAs was performed according to the protocol reported by Messner and Murkovic (2004), incorporating modifications (Oz et al., 2016). The extraction protocol of HAAs is given in Appendix S1. Subsequent solid‐phase extraction, the quantification of HAAs was carried out employing HPLC (Thermo Scientific, USA). A reverse phase Semi Micro ODS‐80 TS column (5 μm, 250 mm × 2 mm i.d.) was employed in the chromatographic analysis (Tosoh Bioscience, Stuttgart, Germany). The separation was carried out employing a mobile phase (eluent A) comprising methanol, acetonitrile, water, and acetic acid (8:14:76:2, respectively). The pH of the mobile phase was adjusted to 5.0 using a 25% ammonium hydroxide solution. Eluent B was composed of acetonitrile. The flow rate was maintained at 0.3 mL/min throughout the analysis. The gradient program was executed as follows: 0% B, 0–12 min; 0%–30% B, 12–20 min; 30% B, 20–35 min. An injection volume of 10 μL was employed, and the column temperature was set at 40°C. Before initiating the analysis, the 4,7,8‐TriMeIQx standard was introduced into the samples via the internal standard addition method. The identification of HAAs was based on the retention times corresponding to the peaks of the established standards. The quantification of HAAs was achieved by calculating the concentrations of HAAs present in the samples, and the results were expressed in ng/g.

Recovery rates of HAAs were calculated by the standard addition method (Kizil et al., 2013). Different concentrations of HAA standards (2.5, 5, 10, and 20 ng/g) were introduced into the raw chicken thigh meat. After the extraction and analysis procedures, the recovery rates were computed. Limit of detection (LOD) and limit of quantification (LOQ) values for HAAs were assessed by the calibration curves derived from standard solutions ranging from 2.5 to 20 ng/g. Method validation parameters (regression coefficient, recovery rates, LOD, LOQ, and relative standard deviations of HAAs) are given in Appendix S1.

### 2.6 Total phenolic content of BME

The quantification of total phenolic content of BME was carried out following the Folin–Ciocalteu method (Hoff & Singleton, 1977). As per the procedure, distilled water and homogenized extract were mixed in glass tubes, then the Folin–Ciocalteu reagent was added, followed by shaking and a 3‐min incubation at room temperature. Afterward, saturated sodium carbonate solution and distilled water were added, and the tubes were placed in darkness for 1 h. The absorbance of the resulting blue‐colored solution was measured at 720 nm to determine the total phenolic content, utilizing a calibration curve with gallic acid standard concentrations, and the results were expressed in milligrams of gallic acid equivalent per kilogram (mg GAE/kg).

### 2.7 Total antioxidant capacity of BME

Total antioxidant capacity of BME was determined by utilizing an ABTS+ radical solution according to the procedure described previously (Serpen et al., 2012). The ABTS solution was prepared by mixing ABTS with deionized water, while the potassium persulfate solution was created by combining potassium persulfate with deionized water. These solutions were then combined to form a stock solution of ABTS+, which was subsequently stored in darkness at room temperature for 12–16 h prior to usage. To achieve the desired absorbance at 734 nm, the stock solution was diluted with a water/ethanol mixture (50:50, v/v) to form the daily working solution of ABTS+. The results were given as millimole Trolox equivalent per kilogram (mmol TE/kg).

### 2.8 Statistical analysis

Factorial analysis of variance was utilized for the statistical analysis in this study. The model integrated the treatment factor, denoted by BME concentration, and cooking temperature as fixed factors. An interaction term was incorporated for the interaction between treatment and cooking temperature. The Tukey's test was employed to ascertain statistically significant differences among groups with more than two categories. Statistical analyses were conducted using SPSS version 23.0.

## 3 RESULTS AND DISCUSSION

### 3.1 Proximate analysis, pH values, and cooking loss of chicken samples

Proximate composition and pH values of raw and cooked chicken samples are given in Table 1. The protein, lipid, moisture, ash content, and pH values of raw chicken samples were 16.95 ± 0.04%, 9.64 ± 0.16%, 73.94 ± 0.47%, 0.91 ± 0.07%, and 6.17 ± 0.99, respectively, aligning with the findings of earlier studies (Gumus & Kizil, 2023; Teng et al., 2022).

**TABLE 1:** Proximate composition, pH values and cooking loss of chicken samples.

	Protein (%)	Lipid (%)	Moisture (%)	Ash (%)	pH	Cooking loss
Raw	16.95 ± 0.04	9.64 ± 0.16	73.94 ± 0.47	0.91 ± 0.07	6.17 ± 0.99	–
EC (%)						
0 (Control)	19.71 ± 2.90	7.93 ± 1.44	64.75 ± 1.39	1.32 ± 0.30	6.79 ± 0.10	31.5 ± 6.1
0.5	19.05 ± 2.61	7.81 ± 2.55	64.16 ± 2.09	1.45 ± 0.25	6.73 ± 0.07	31.1 ± 5.6
1	19.64 ± 2.60	6.99 ± 2.39	64.79 ± 1.99	1.28 ± 0.18	6.82 ± 0.10	32.1 ± 6.0
Significance	ns	ns	ns	ns	ns	ns
CT (°C)						
150	19.10 ± 3.15	7.94 ± 1.55	66.48 ± 2.02^a^	1.23 ± 0.12^a^	6.80 ± 0.09	24.9 ± 1.1^a^
200	20.76 ± 2.53	6.98 ± 2.47	65.12 ± 2.23^b^	1.33 ± 0.18^ab^	6.79 ± 0.10	33.9 ± 1.3^b^
250	19.47 ± 2.16	8.24 ± 1.37	63.71 ± 1.34^c^	1.44 ± 0.22^b^	6.79 ± 0.11	37.8 ± 1.0^c^
Significance	ns	ns	*	*	ns	**
EC × CT	*	*	ns	*	ns	ns

*Note*: The values are given as mean ± standard deviation. *p < .05, **p < .01. Results with different letters (a–c) in the same column are significantly different.

Abbreviations: CT, cooking temperature (°C); EC, bitter melon extract concentration (%); ns, not significant.

The cooking process has influenced both the proximate results and pH values, and the findings were in line with the results reported in various other studies (Gumus & Kizil, 2023; Kwon et al., 2023). The process of cooking resulted in a change in both protein and lipid content, a phenomenon that might be linked to the decrease in moisture content and cooking loss (Teng et al., 2022). The cooking loss of samples ranged between 23.2 and 39.3%. Notably, the increase in the ash content and cooking loss between samples cooked at varying temperatures exhibited statistical significance (*p* < .05). Additionally, the impact of BME on the proximate results, pH values, and cooking loss of cooked chicken samples was not statistically significant (*p* > .05). The predominant factor influencing these results was the cooking process and cooking temperature, consistent with findings from prior studies (Haskaraca et al., 2014; Lu et al., 2018).

### 3.2 HAA levels of cooked chicken samples

Based on our current knowledge, this is the initial research investigating the impact of the marinades of BME on HAA formation in chicken thigh meat. Various approaches, including modifying cooking parameters (such as cooking methods, temperature, and duration), utilizing marinades and incorporating spices, and synthetic and natural extracts have been explored to mitigate the generation of HAAs (Dong et al., 2020; Kang et al., 2022). Marinating meat products before cooking has proven to be one of the most efficient methods for minimizing the overall generation of HAAs, and this has been attributed to the combined physical and chemical influences of marinades on the Maillard reaction, which is responsible for HAA generation (Khan, Khan, et al., 2022). HAA contents of chicken samples are presented in Table 2. Among the 10 different HAAs analyzed, Trp‐P‐2 was not found in any of the samples. Likewise, Trp‐P‐2 was not detected in previous investigations in chicken samples cooked at 215°C (Khan, 2015) or in pork samples cooked at 180°C (Kwon et al., 2023). Notably, the other HAAs were present at levels that varied according to the cooking temperature and BME concentration in the present study. The formation of HAAs at varying levels depending on the cooking temperature and the presence of marinade is consistent with previous studies (Gumus & Kizil, 2022; Hasnol et al., 2014; Khan, Luo, et al., 2022).

**TABLE 2:** HAA levels of chicken samples (ng/g).

CT	EC	IQx	IQ	MeIQx	MeIQ	7,8‐DiMeIQx	4,8‐DiMeIQx	Trp‐P‐2	PhIP	AαC	MeAαC	Total HAAs	Inhibition (%)
150°C	Control	0.03	0.16 ± 0.10	0.28	0.27 ± 0.05	0.58	0.12	nd	nd	nd	nd	1.73 ± 1.89	
	0.5%	0.01	0.07 ± 0.04	0.07	nd	0.08 ± 0.01	0.06	nd	nd	nd	nd	0.69 ± 0.69	−60.1
	1%	0.02	0.07	nd	nd	0.43	nd	nd	nd	nd	nd	0.52 ± 0.73	−69.9
200°C	Control	0.27 ± 0.07	0.16 ± 0.07	0.44 ± 0.16	0.39	1.09 ± 0.94	0.19 ± 0.07	nd	nd	0.82 ± 0.74	nd	5.49 ± 5.61	
	0.5%	0.25 ± 0.23	0.40 ± 0.15	0.28 ± 0.04	nd	0.76 ± 0.00	0.19 ± 0.01	nd	0.28	0.32	nd	4.07 ± 1.48	−25.9
	1%	0.04	0.10 ± 0.08	0.21	nd	0.73 ± 0.16	nd	nd	nd	0.27	nd	1.88 ± 1.37	−65.8
250°C	Control	0.31 ± 0.33	1.00 ± 1.27	0.50 ± 0.05	1.02	1.98 ± 0.71	0.20	nd	0.83	nd	nd	8.94 ± 2.00	
	0.5%	0.24 ± 0.07	0.19 ± 0.08	0.26 ± 0.17	0.49 ± 0.33	0.89 ± 0.41	0.23 ± 0.08	nd	0.85	0.54 ± 0.12	0.59	6.04 ± 4.83	−32.4
	1%	0.06 ± 0.05	0.14 ± 0.06	0.22 ± 0.14	0.25 ± 0.29	1.08 ± 0.55	0.19 ± 0.15	nd	nd	0.22	nd	3.76 ± 1.33	−57.9

*Note*: Values are expressed as mean or mean ± standard deviation. −, decrease in formation of total HAAs compared to control groups.

Abbreviations: CT, cooking temperature (°C); EC, bitter melon extract concentration (%); nd, not detected.

Varying amounts of IQx were formed in the samples, reaching up to 0.55 ng/g. Among the samples at each cooking temperature, the control groups consistently contained the highest levels of IQx. In general, an increase in cooking temperature elevated IQx levels in the control samples, consistent with previous reports in prior studies (Asmaa'Ishak et al., 2022; Fan et al., 2022). Notably, samples marinated with BME exhibited lower IQx levels. Similarly, marinades containing various levels of bilberry extract decreased the generation of IQx in chicken thigh samples pan‐fried at 150 and 200°C (Gumus & Kizil, 2022).

The highest level of IQ detected among the analyzed samples was 1.90 ng/g, observed in the control samples cooked at 250°C. Overall, marination with BME resulted in a decrease in IQ levels compared to the control groups. In an earlier research, green, white, and oolong tea marinades exhibited mitigative effects on the generation of IQ in grilled chicken samples (Yao et al., 2020). Conversely, among the samples cooked at 200°C, the IQ level of the sample containing 0.5% BME was higher than that of the control group in the present study. Similarly, in a previous investigation, marinades containing table sugar were found to elevate the levels of IQ formation in grilled chicken samples (Hasnol et al., 2014). Different findings have been documented regarding the impact of various cooking conditions on the IQ levels of chicken meat (Caliskan et al., 2023; Hasnol et al., 2014). Additionally, it has been suggested that the inhibitory and enhancing impacts of antioxidant compounds on HAAs generation may not necessarily correspond with their antioxidant capabilities, as antioxidants can exhibit pro‐oxidant properties depending on their concentration (Ekiz et al., 2023; Zeng et al., 2017).

The highest MeIQx level among all samples was determined as 0.56 ng/g. Similarly, MeIQx levels were up to 0.63 ng/g in chicken thigh samples pan‐fried at 150, 200, and 250°C for 14 min (Caliskan et al., 2023). Notably, increasing the cooking temperature tended to raise MeIQx levels in the current study. Samples marinated with BME exhibited reduced levels of MeIQx compared to their respective control samples. A similar inhibitory effect was noted in an earlier research, where MeIQx contents of chicken samples were decreased by the addition of perilla frutescens seed extract (Khan, Luo, et al., 2022).

MeIQ was found to be present at 1.02 ng/g in the sample which had the highest formation levels. Marination with BME elicited a complete inhibition of MeIQ formation in samples subjected to cooking temperatures of 150 and 200°C. Additionally, BME marinades have demonstrated their effectiveness in reducing MeIQ levels when compared to the control group in the samples cooked at 250°C. In a comparable manner, marinades of blueberry, raspberry, and strawberry extracts mitigated the levels of MeIQ in pan‐fried chicken samples in a previous study (Khan et al., 2021). Another prior investigation demonstrated that extracts from olive and lotus leaves decreased the MeIQ levels in pan‐fried chicken breast samples (Dong et al., 2011).

The highest level of 7,8‐DiMeIQx was quantified as 2.48 ng/g in the control sample cooked at 250°C. Overall, samples cooked at higher temperatures had higher levels of 7,8‐DiMeIQx. Furthermore, marinating chicken thigh meat with BME reduced the formation of 7,8‐DiMeIQx at all cooking temperatures. Similarly, marinades containing varying concentrations of bilberry extract (Gumus & Kizil, 2022) and the addition of rosa rugosa tea extract (Jamali et al., 2016) mitigated the generation of 7,8‐DiMeIQx levels in different meat types.

In the present research, 4,8‐DiMeIQx was detected at varying levels up to 0.30 ng/g. Among the samples cooked at 150 and 200°C, those containing BME marinades had lower levels of 4,8‐DiMeIQx compared to the control groups. A previous study demonstrated that the inclusion of garlic, onion, red chili, paprika, ginger, and black pepper powder had a diminishing impact on the levels of 4,8‐DiMeIQx in fried chicken meatball samples (Lu et al., 2018). Conversely, in the current study, samples marinated with 0.5% BME exhibited elevated 4,8‐DiMeIQx levels. Comparable findings regarding 4,8‐DiMeIQx levels have been reported in previous studies, and these observations might be attributed to the dual oxidative and antioxidative properties of compounds, contingent on their concentration (Caliskan et al., 2023; Zeng et al., 2016).

PhIP stands out as the predominant HAA generated during the cooking of meat. However, no PhIP formation was observed in samples cooked at 150°C. PhIP is recognized for its tendency to form at elevated temperatures (Dong et al., 2020; Zöchling & Murkovic, 2002). Therefore, lack of PhIP formation at 150°C in the present study might be attributed to various factors including the precise cooking conditions and duration. Notably, among samples cooked at 200°C, only those marinated with 0.5% BME contained PhIP. Furthermore, while PhIP was present in samples marinated with 0% and 0.5% BME, the presence of 1% BME inhibited PhIP formation at 250°C. In a similar vein, PhIP was absent in meatball samples cooked at 150°C but was detected at temperatures of 200 and 250°C in an earlier research (Ekiz et al., 2023). Likewise, other previous studies indicated that artichoke extract (Tengilimoglu‐Metin & Kizil, 2017) and *Vaccinium myrtillus* L. extract (Gumus & Kizil, 2022) marinades had varying effects on the PhIP levels of pan‐fried chicken breast and chicken thigh samples, respectively, contingent on the concentration and cooking conditions.

The outcomes regarding the impacts of temperature and BME on AαC exhibited variability. For instance, AαC formation did not occur in the samples cooked at 150°C. Significantly, AαC formation amounted to 0.82 ± 0.74 ng/g in the control samples cooked at 200°C, while in the samples marinated with BME, its formation level decreased compared to the control samples. On the other hand, while no AαC formation was observed in the control group of samples cooked at 250°C, AαC formation was detected in the samples marinated with BME. In a comparable manner, among all samples, MeAαC was only detected in the sample cooked at 250°C with 0.5% BME, while it was not observed in the other samples. Various marinades might exert varying impacts on the generation of HAAs in meat products, and these effects might vary depending on the preparation and cooking conditions, composition, and concentration (Gumus & Kizil, 2022; Yao et al., 2020; Zeng et al., 2016). For instance, in a previous study, the oolong tea marinade increased the AαC levels of grilled chicken samples, while white and green tea marinades exhibited inhibitory effects (Yao et al., 2020).

While BME generally demonstrated a reduction in the formation of HAAs in this study, it was noted that the marinades containing BME had dose‐dependent impacts on the generation of certain HAAs at specific temperatures. Numerous reports in the literature are in line with this finding (Gumus & Kizil, 2022; Zeng et al., 2017; Zhao et al., 2023). In an earlier study that investigated the inhibitory effects of cyanidin at varying concentrations (0.25%, 0.5%, 1.0%, or 2.0% w/v) on the formation of PhIP, Harman, and Norharman in smoked chicken drumsticks, cyanidin demonstrated a dose‐dependent and effective inhibition of HAAs generation (Zhang et al., 2023).

Total HAA levels of control samples were 1.73 ± 1.89, 5.49 ± 5.61, and 8.94 ± 2.00 ng/g in chicken samples cooked at 150, 200, and 250°C, respectively. Notably, samples marinated with BME had lower total HAA contents compared to the control groups at all temperatures. In a prior investigation, the levels of total HAAs in roasted chicken meat, prepared at various cooking durations (30, 50, 70, 90, 110, and 130 min) and temperatures (175, 200, 225, and 250°C), varied between 5.04 and 39.59 ng/g (Yao et al., 2023). In another earlier study, the total HAA levels in roasted chicken exhibited a range of 0.25–2.41 ng/g (Hsu & Chen, 2020).

**FIGURE 1:**
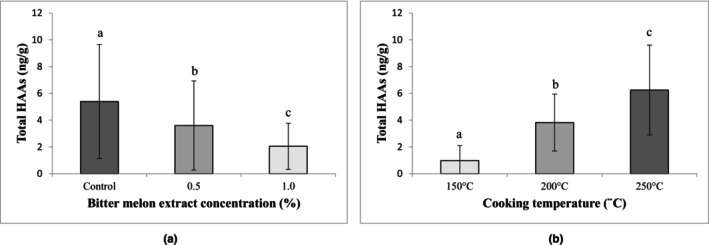
Levels of total HAAs according to the extract concentration (a) and cooking temperature (b) (ng/g). The means are given with standard deviation. Results with different letters (a–c) in the same figure are significantly different (*p* < .05).

Levels of total HAAs according to the extract concentration and cooking temperature are given in Figure 1. Notably, a significant difference was observed between samples cooked at various temperatures. Chicken thigh meat cooked at 150, 200, and 250°C had 0.98 ± 1.12, 3.82 ± 2.12, and 6.25 ± 3.35 ng/g total HAAs, respectively (*p* < .01). Furthermore, the addition of BME to the marinades had a notable effect on the total HAAs (*p* < .05). The total content of HAAs in the control samples was 5.39 ± 4.26 ng/g, whereas, in comparison, samples marinated with 0.5% and 1% BME exhibited 3.60 ± 3.33 and 2.05 ± 1.72 ng/g total HAAs, respectively. The reduction rates of total HAAs with BME were significantly higher when using 1% BME compared to 0.5% BME. Nonetheless, using BME in the marinades led to a 33.2% reduction in total HAAs with 0.5% BME and a 62% reduction with 1% BME. In a previous investigation, the inclusion of *Adinandra nitida* leaf extract resulted in a reduction of total HAA levels in deep‐fried chicken samples ranging from 11.98% to 38% (Zhao et al., 2023). In another prior study, the addition of quercetin led to a decrease in total HAA levels in chicken drumsticks deep‐fried at 180°C (Li et al., 2023). Marinating chicken thigh samples with propolis extract resulted in a decrease of total HAA levels by 41.2%–89.4% at 150°C and 49.4%–91.4% at 200°C in a previous research (Gumus & Kizil, 2023).

BM contains phenolic compounds demonstrating potent antioxidant properties (Budrat & Shotipruk, 2008). The predominant phenolic acids present in BM were reported as gallic acid, gentisic acid, catechin, chlorogenic acid, and epicatechin (Horax et al., 2005; Psilopatis et al., 2023). The inhibitory impacts of BME on HAA generation might be ascribed to the total antioxidant capacity and phenolic content of the extract, which were measured as 150.8 ± 24.33 mmol TE/kg and 35035.71 ± 893 mg GAE/kg, respectively. Consistent with the current results, previous research also demonstrated that BME exhibited a high antioxidant capacity and phenolic content (Akyuz et al., 2020; Lee et al., 2017; Tan et al., 2014). For instance, the total phenolic content and antioxidant capacity of aqueous and ethanolic BME were measured as 10.6 ± 0.2 mg GAE/g dw and 94.8 ± 1.3 μmol TE/g, and 10.7 ± 0.3 mg GAE/g dw and 95.7 ± 3.5 μmol TE/g, respectively (Tan et al., 2014).

Dietary HAAs are known to undergo metabolic activation in the human body, leading to the formation of DNA adducts that can induce mutations and potentially contribute to the development of various cancers, particularly in organs such as the colon, prostate, and breast (Oz et al., 2023). The International Agency for Research on Cancer (IARC) has identified more than 10 HAAs as possible human carcinogens and one HAA (IQ) as a probable human carcinogen (Dong et al., 2020; Geng et al., 2023). HAAs have been associated with adverse effects on human health, highlighting the importance of understanding their mechanisms of action and implementing strategies to mitigate their formation in cooked foods (Geng et al., 2023). While the precise mechanisms of the generation of HAA in meat remain partially elucidated, extant understanding indicates that free radicals play a central role. Thus, it is suggested that antioxidants may reduce HAA production by impeding various stages of free radical generation, a process pivotal in HAA generation (Dong et al., 2020; Khan, Khan, et al., 2022; Oz et al., 2023). Therefore, the capacity of BME in our study to reduce HAA levels could be linked to its ability to delay or mitigate lipid oxidation in meat, as suggested by associations between oxidation and increased HAA formation, along with its potential interception of crucial intermediates in the HAA generation pathway. It is noteworthy that the influence of antioxidants can exhibit variability, potentially acting as prooxidants contingent upon factors including molecular structure, dosage, substrate, application conditions, temperature, and other pertinent variables (Geng et al., 2023; Uzun & Oz, 2021).

Despite offering valuable insights into mitigating the formation of HAAs in chicken thigh meat, some limitations should be acknowledged. The findings of this study are specific to the experimental conditions employed, including the type of meat, cooking methods, and concentrations of BME used. Additionally, while BME demonstrated inhibitory effects on HAA formation, the specific mechanisms underlying these effects remain to be fully elucidated. Finally, although the researchers conducted preliminary sensory evaluations to ensure the general acceptability of the samples, it is important to note that consumer acceptance of chicken products marinated with BME was not directly assessed in this study. While efforts were made to establish general sensory acceptability, individual consumer preferences might vary, and factors such as cultural background, taste preferences, and familiarity could influence consumer perceptions.

## 4 CONCLUSION

This research is the initial examination of the impact of BME on HAA formation in chicken meat as far as we know. In brief, higher levels of total HAAs were generated at elevated cooking temperatures, with variations in the types and quantities of HAAs depending on the temperature and the levels of BME. Marination with BME was found to mitigate the total HAA levels in cooked chicken samples between 25.9% and 69.9%, and the extent of inhibition was contingent on the concentration of the extract and cooking temperature. The results obtained from this study hold significant potential for the integration of natural extracts into chicken products as a viable strategy for the inhibition of HAAs. Further investigations could focus on elucidating the effects of BME on other carcinogens that occur in cooked meat, as well as optimizing the concentration and application methods of the extract to maximize its efficacy. Additionally, future research efforts might explore the consumer acceptability of chicken products marinated with BME to ensure their feasibility and marketability.

## AUTHOR CONTRIBUTIONS

**Damla Gumus:** Data curation (equal); formal analysis (equal); investigation (equal); writing – original draft (equal). **Arife Macit:** Data curation (equal); formal analysis (equal); investigation (equal). **Bengu Guzel:** Formal analysis (equal); investigation (equal). **M. Merve Tengilimoglu‐Metin:** Investigation (equal). **Mevlude Kizil:** Conceptualization (lead); investigation (equal); methodology (lead); project administration (lead); resources (lead); supervision (lead); validation (lead); writing – review and editing (lead).

## Supporting information


Appendix S1

## Data Availability

The data that support the findings of this study are available from the corresponding author upon reasonable request.
