# Supplementary material for: Bitter melon extract mitigates heterocyclic aromatic amine formation in chicken thigh meat
Source: Food Sci Nutr. 2024 Mar 7;12(6):4259–68. doi: 10.1002/fsn3.4085 (PMC11167177; doi:10.1002/fsn3.4085)
Supplement: Supplementary file 1 — Appendix S1 [file FSN3-12-4259-s001.docx]

**SUPPLEMENTARY MATERIAL**

**Bitter Melon Extract Mitigates Heterocyclic Aromatic Amine Formation in Chicken Thigh Meat**

Damla Gumus^a^, Arife Macit^a^, Bengu Guzel^a^, M. Merve Tengilimoglu-Metin^a^, Mevlude Kizil^a*^

^a^Department of Nutrition and Dietetics, Faculty of Health Sciences, Hacettepe University, 06100, Sihhiye, Ankara, Turkey

* Corresponding author:

Mevlude Kizil, mkizil@hacettepe.edu.tr (0000-0003-1380-3243)

**Extraction of HAAs**

HAAs were extracted from the chicken samples following the procedure as follows: 1 g of the sample was dissolved in 12 mL 1 M NaOH. The resulting suspension was homogenized using magnetic stirring at 500 rpm for 1 h at room temperature. The solution was combined with 13 g diatomaceous earth (Extrelut NT Packaging Material, Merck, Darmstadt, Germany) and then transferred into empty Extrelut columns. Ethyl acetate was utilized for the extraction, and the eluate was passed through coupled Oasis MCX cartridges. The cartridge underwent washing with 2 mL 0.1 M HCl and 2 mL MeOH. The analytes were eluted using 2 mL MeOH-ammonia solution (25% v/v, 19:1). The eluted mixtures were evaporated to dryness at 50°C. The resultant extracts were dissolved in 100 μL MeOH before HPLC measurement.

**Supplementary Table.** Regression coefficient, recovery rates (%), limit of detection, limit of quantification, and relative standard deviations of HAAs

| **HAA** | **Regression coefficient (R^2^)** | **Recovery (%)** | **LOD (ng/g)** | **LOQ (ng/g)** | **Intra-day RSD (%)** | **Inter-day RSD (%)** |
| --- | --- | --- | --- | --- | --- | --- |
| **IQx** | 0.9979 | 78.20 | 0.002 | 0.006 | 10.08 | 14.79 |
| **IQ** | 0.9994 | 77.37 | 0.002 | 0.005 | 8.81 | 11.48 |
| **MeIQx** | 0.9966 | 71.78 | 0.003 | 0.010 | 12.48 | 13.68 |
| **MeIQ** | 0.9923 | 80.60 | 0.004 | 0.012 | 13.69 | 17.17 |
| **7,8-DiMeIQx** | 0.9954 | 74.82 | 0.002 | 0.006 | 6.33 | 8.92 |
| **4,8-DiMeIQx** | 0.9981 | 75.22 | 0.003 | 0.008 | 16.61 | 18.89 |
| **Trp-P-2** | 0.9983 | 78.48 | 0.004 | 0.011 | 13.34 | 14.23 |
| **PhIP** | 0.9977 | 84.39 | 0.004 | 0.013 | 9.76 | 16.05 |
| **AαC** | 0.9936 | 73.13 | 0.005 | 0.014 | 6.65 | 14.18 |
| **MeAαC** | 0.9957 | 74.51 | 0.004 | 0.013 | 7.93 | 9.65 |

LOD; limit of detection, LOQ; limit of quantification, RSD; relative standard deviation.
